# Hansenula Polymorpha TERT:  A Telomerase Catalytic Subunit Isolated in Recombinant Form with Limited Reverse Transcriptase Activity

**Published:** 2012

**Authors:** E.M. Smekalova, O.A. Petrova, M.I. Zvereva, O.A. Dontsova

**Affiliations:** Chemistry Department, Lomonosov Moscow State University

**Keywords:** telomerase reverse transcriptase, recombinant proteins, thermotolerant yeast*Hansenula polymorpha.*

## Abstract

Telomerase is a ribonucleoprotein, the main function of which is to synthesize telomeres, i.e. repetitive sequences which are localized at the ends of eukaryotic chromosomes. Telomerase maintains the stability of the genome in eukaryotic cells by replicating chromosomal ends. The structural and functional investigation of the telomerase complex is significantly restricted due to difficulties connected with the isolation of its main catalytic subunit in recombinant form. Herein, we describe a method developed for the isolation of the recombinant telomerase reverse transcriptase from thermotolerant yeast*Hansenula polymorpha*. A functional test performed for the isolated protein and the RNA/DNA duplex, simulating the interaction of telomerase RNA and telomere, reveals that the isolated catalytic subunit of telomerase possesses limited reverse transcriptase activity.

## INTRODUCTION

Telomerase is a ribonucleoprotein, the main function of which is to synthesize telomeres, i.e. repetitive sequences that are localized at the ends of eukaryotic chromosomes, unable to replicate in accordance with the classical replication mechanism. Telomerase exhibits activity in cells capable of infinite division, such as germinal and stem cells, as well as in the majority of malignant tumors (85%). It is believed that the inhibition of the telomerase catalytic function halts the maintenance of the telomere length, thereby eliminating the infinite replication potential of tumor cells. Altogether, it allows telomerase to be considered as a universal target for various antitumor drugs [1]. The main components of telomerase are the protein, the telomerase reverse transcriptase (TERT), and the telomerase RNA, the matrix of which serves as a template for the synthesis of the telomere sequence [2]. One of the main difficulties in studying telomerase is the low stability of its catalytic subunit isolated in recombinant form [3]. The absence of data on the structure of telomerase prevents the docking of known substances with a view to searching for the potential effectors of this enzyme, as well as the difficulties attached to isolating the full-length functional telomerase reverse transcriptase, which thus impede the testing of the interactions between pharmacological agents and the target. At the time of writing, the TERT isolated from * Tribolium castaneum* is the only full-length telomerase reverse transcriptase that has been isolated [4]. The distinctive feature of this protein is the absence of the N-terminal domain typical for other telomerase reverse transcriptases. Data on the structure of the N-terminal domain in the telomerase catalytic subunit of *Tetrahymena thermophile* and its RNA-binding domain have been obtained [2, 5].

The use of thermophilic organisms is promising for structural and functional studies of proteins, since these organisms possess a more compact spatial organization, which facilitates its stabilization in a solution. Earlier, we identified the telomerase reverse transcriptase of thermotolerant yeast *Hansenula polymorpha* (hpTERT) and have shown for the first time that hpTERT can be expressed in the cells of *Escherichia coli* and that the recombinant protein can be isolated [6]. We have developed a method for the effective isolation of hpTERT expressed in *E.*   *coli* . Specific expression constructions were used, enabling the production of the telomerase catalytic subunit with various affinity tags at either the C- or N-terminus of the protein. It was shown that the optimal vector for the expression and isolation of hpTERT is pET30aTEV, in which the open reading frame encodes hpTERT with 6His- and S-tags at the N-terminus. A test was performed that served to confirm the exhibition of reverse transcriptase activity by this protein, thus proving that it is suitable for functional and structural studies. We believe that this report will be useful not only for researchers who study telomerase, but also for those who face problems in obtaining recombinant proteins that are unstable in their soluble form.

## EXPERIMENTAL

**Cloning of the *hpTERT* Gene into Various Expression Systems **

The *hpTERT* gene was cloned using the following primers: 1) BamH1a/E2 (5’-aaggatccaaggtttgatcagtatgttgatga-3’) and E2/Pst1/Rev (5’-tttctgcagttagaatgctttaagaagcga-3’) for obtaining the pCDF plasmid, in which hpTERT is fused with a 6His tag at the N-terminus; 2) Nco1E2Fwd (5’-aaaaaccatgggaaggtttgatcagtatgttgat-3’) and E2Sal1Rev (5’-tttttgtcgac gaatgctttaagaagcgaac-3’) for obtaining pET33b+, in which hpTERT is merged with a 6His tag at the С-terminus; 3) HpET30F (5’-gacggagctcgaattttattagaatgctttaagaagcgaac-3’) and HpET30S (5’-gtattttcagggcgccatgaggtttgatcagtatgttgat-3’) for obtaining the pET30aTEV, in which hpTERT is merged with 6His- and S tags at the N-terminus. The pET30aTEV was kindly provided by Daniela Rhodes (MRS LMB, Cambridge, United Kingdom). DNA sequencing was carried out using a set of ABI PRISM® BigDye ^TM^ Terminator v. 3.1 reagents, followed by an analysis of reaction products by means of an Applied Biosystems 3730 DNA Analyzer.

**Isolation and Purification of Recombinant hpTERT**

*E. coli* BL21DE3 cells transformed with either a pCDF_hpTERT plasmid or pET33b_hpTERT or pET30_hpTERT were cultivated at 37°C until an optical density of 0.1–0.3 ( *OD*_600_ ); following this, the expression of the protein was induced by 0.1 mM isopropyl-thio-β-D-galactoside (IPTG) and the mixture was incubated upon stirring for 12–16 h at 16°C. The cells were collected via centrifugation at 5000 rev/min and cooled by liquid nitrogen; they were then disintegrated using a dismembrator (2000 rev/min, twice, for 30 sec each); the latter provided less denaturation of the protein during the isolation process. Disintegrated cells were re-suspended in buffer A: 50 mM NaH _2_ PO _4_ (pH 7), 200 mM NaCl, 10% glycerol, 10 mM β-mercaptoethanol, and 0.05% Tween-20. Cell debris were separated by centrifugation at 15000 rev/min for 20 minutes. A cell lysate was then incubated with Ni-NTA-agarose for 30 min at 4°C; an affinity sorbent was separated from the unbound protein fraction by centrifugation at 3000 rev/min, followed by decantation of the supernatant. Ni-NTA-agarose was washed three times with buffer A containing 50 mM imidazole. The hpTERT protein bound to the affinity sorbent was eluted with buffer A containing 300 mM imidazole.

During additional purification by ion-exchange chromatography on SP-sepharose, the sample obtained in the previous stage was diluted to a total concentration of salts of 150 mM, and SP-sepharose pre-equilibrated in buffer B (50 mM NaH _2_ PO _4_ (pH 7), 100 mM NaCl, 10% glycerol, 10 mM β-mercaptoethanol, and 0.05% Tween-20) was added. The bound protein fraction was washed away using a gradient of NaCl concentration (0.1–1 M) in an analogous buffer. Glycerol (up to 30%) was added to the sample; the latter was subsequently frozen under liquid nitrogen and stored at –80°C.

**Testing of the Functionality of Purified hpTERT **

*in vitro*

The functionality of hpTERT was tested in a system containing 50 mM Tris-HCl, 1 mM DTT (dithiothreitol), 1 mM spermidine, 50 µM dCTP, 5 µM substrate (5’-cgccaccccgccaccc-3’ RNA oligonucleotide and 5’-cgccaccccgccaccc-3’, 5’-ggcgggcggggtg-3’ DNA oligonucleotides were used), 3.75 µM [α- ^32^ P] dGTP (800 Cu/mmol), and 5 µM hpTERT. Duplexes (DNA-DNA or DNA-RNA) were formed by hybridization of corresponding oligonucleotides. The reaction was performed at a temperature of 37°C for 30 min; the mixture was then treated with protein kinase K (0.3 mg/ml) and re-precipitated in alcohol. The products of the reaction were separated by gel electrophoresis in a 15% denaturing polyacrylamide gel (PAAG). Radioactive signals were detected by means of the Phosphorimager system.

## RESULTS AND DISCUSSION

**Fig. 1:**
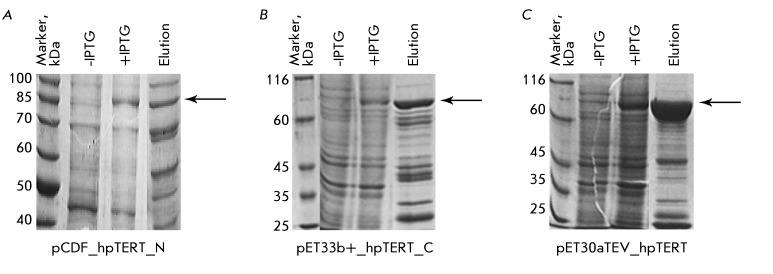
Results of the expression and affinity isolation of recombinant hpTERT from *E. coli* cells transformed with different plasmids: *A* – the hpTERT is cloned into the pCDF vector with a 6His tag at its N-terminus; *B*  – hpTERT is cloned into the pET33b+ vector with a 6His tag at its C-terminus; *C*  – hpTERT is cloned into the pET30aTEV vector with 6His- and S tags at its N-terminus. The samples of *E. coli* cells before and after IPTG induction of expression and the sample of elution of hpTERT from Ni-NTA agarose were analyzed by denaturing PAGE electrophoresis. The zone corresponding to the mobility of the hpTERT protein in the gel is indicated by an arrow.

**Fig. 2:**
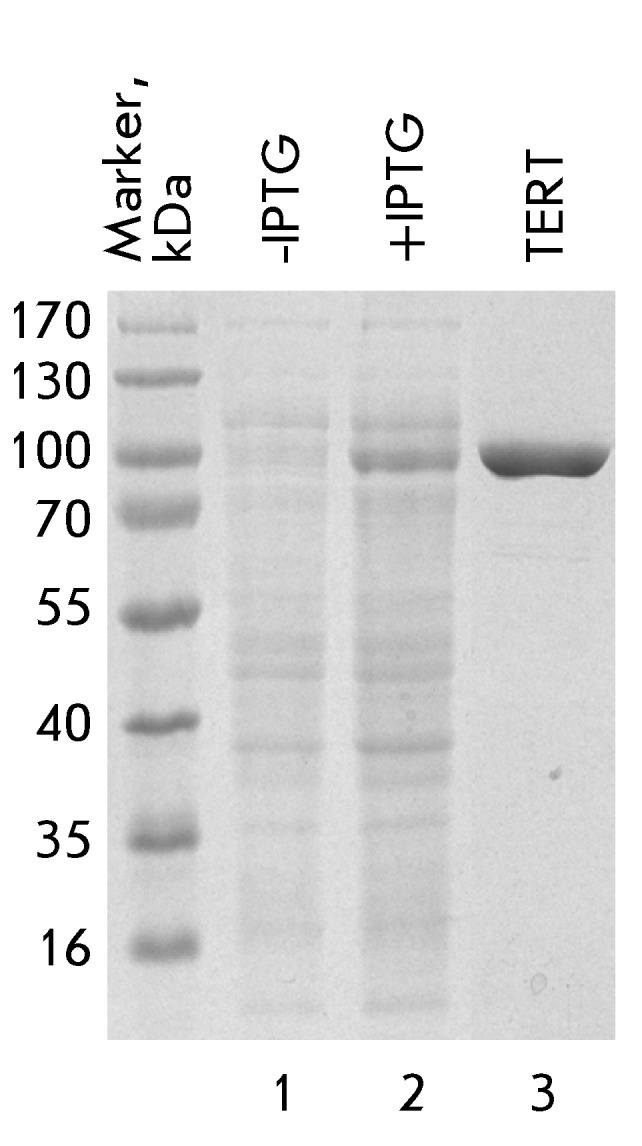
The expression, isolation, and purification of hpTERT. Gel electrophoresis of the proteins from *E. coli* cells transformed with plasmid pET30aTEV_hpTERT without IPTG induction (-IPTG) and after IPTG induction of the hpTERT expression (+IPTG). The hpTERT sample corresponds to the protein obtained by affinity purification of cell lysate, followed by ion-exchange chromatography.

The gene of the hpTERT protein was cloned under the control of the T7 promotor into the following three expression systems, with the purpose of further isolation of the protein from *E. coli* cells: 1) pCDF with a 6His tag at the N-terminus of the hpTERT protein; 2) pET33b+ with a 6His tag at the С-terminus of the hpTERT protein; and 3) pET30aTEV with 6His- and S tags at the N-terminus of the hpTERT protein. Such location of tags, *i.e.* from different sides of the protein, relates to the ability of the amino acid sequence termini to fold inside a protein globule; the latter is most probably one of the reasons behind the decrease in the effectiveness of affinity chromatography. The S tag is a short sequence (4 kDa) which can be used for the stabilization of proteins in a solution. The expression of proteins was induced by IPTG; the proteins were purified by metal-chelate chromatography on Ni-NTA-aragose. The results on the isolation of the proteins expressed by using various constructions are shown in *Fig. 1* . The hpTERT protein is detected in all the samples eluted with Ni-NTA-agarose. The latter indicates that the selection of thermotolerant yeast as a source for the production of the telomerase catalytic subunit was successful. However, in the cases occurring when the pCDF and pET33b+ vectors are used and a tag is located at the N-terminus or C-terminus or at both ( *Figs. 1A, 1B* ), a significant amount of impurities are detected on the resin, along with a target protein. It should be noted that the amount of impurities relative to the amount of the target protein is lower when the pET33b+ with a 6His tag at the C-terminus of the protein is used; in all likelihood, this reflects the close orientation of the N-terminus in hpTERT. The isolation of the protein with the help of the S tag (the pET30aTEV construction) gives a much better result ( *Fig. 1C* ). Apparently, a well-structured short N-terminal S tag significantly enhances the stability of the protein soluble form. The protein was isolated and additionally purified by ion-exchange chromatography on SP-sepharose ( *Fig. 2* ). The final characteristics of the protein sample obtained are the following: the concentration is 5 mg/ml, the yield is 5 mg/L of the *E. coli* cell culture, and the content of impurities is not more than 1%.

**Fig. 3:**
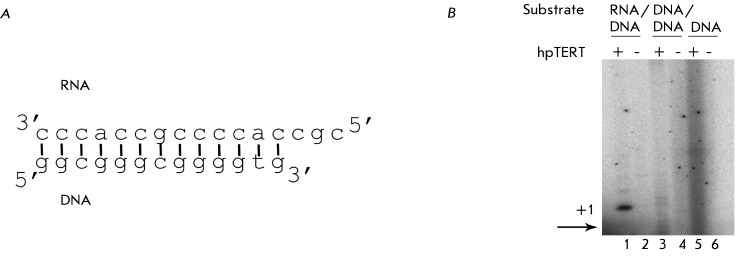
Recombinant hpTERT isolated from *E. coli* cells transformed with the pET30aTEV vector acts as a reverse transcripatase. *A – * Scheme of the RNA/DNA duplex, which is used as a substrate for hpTERT; *B – * Products of the reaction of hpTERT (the presence of the protein is marked by +) and different substrates (the type of the used substrate is indicated above the picture). Products of the reaction are visualized via the introduction of radioactively labeled dGTP into the reaction mixture. The zone which corresponds to the mobility of the initial DNA oligonucleotide is marked by an arrow. The primer extension is present only in the first lane; this proves that recombinant hpTERT acts as a reverse transcriptase in this model system.

The functionality of the obtained protein was confirmed with the help of a constructed *in vitro* system. *H. polymorpha* telomeres consist of 18–23 repeats (5’-GGGTGGCG-3’) [7]. On the basis of these data, a composition of the telomerase RNA fragment can be suggested and the DNA oligonucleotide representing a telomere can be modeled. Thus, the system contained the purified recombinant telomerase catalytic subunit, the substrate that is a hybrid RNA/DNA duplex with a free 3’-terminus ( *Fig. 3A* ), the mixture of nucleotides in which [α- ^32^ P]dGTP was used for the visualization of oligonucleotide elongation. Either the analogous DNA/DNA duplex or the single-stranded telomerase DNA was used as a control. Since the telomerase catalytic subunit is a reverse transcriptase, such substrates cannot be used by hpTERT for elongation. Moreover, each reaction was performed in the presence and the absence of hpTERT ( *Fig. 3B* ). In lane *1* ( *Fig. 3B* ), a specific signal corresponding to the binding of dGTP to the DNA oligonucleotide in the RNA/DNA duplex can be observed. This zone is absent in systems with other substrates and in the absence of the protein, thereby eliminating the participation of the *E. coli* polymerases in this reaction. The RNA/DNA duplex used in this reaction was constructed so that the binding of three nucleotides was possible in the system. The signals corresponding to the binding of the second and third nucleotides can be seen in lane *1* ( *Fig. 3B* ), despite the fact that their intensity is much lower. In all likelihood, this is associated with the absence of the full-length telomerase RNA in the system, which is required for the reconstruction of telomerase activity *in vitro* . Nevertheless, the binding of even one nucleotide indicates that the protein has a specific reverse transcriptase activity, and that its functional structure is preserved.

Thus, the pET30aTEV construction with hpTERT, in which the 6His- and S-tags are located at the N-terminus of the protein, can be used for the production of the recombinant functional catalytic *H. polymorpha* telomerase subunit. This opens up new opportunities for the determination of the structure of the telomerase reverse transcriptase and the study of its functional mechanism. 
